# *SIRT1* and *FOXO1* role on MASLD risk: effects of DHA-rich n-3 PUFA supplementation and exercise in aged obese female mice and in post-menopausal overweight/obese women

**DOI:** 10.1007/s13105-024-01044-9

**Published:** 2024-09-12

**Authors:** Jinchunzi Yang, Elisa Félix-Soriano, Alejandro Martínez-Gayo, Javier Ibañez-Santos, Neira Sáinz, J Alfredo Martínez, María J. Moreno-Aliaga

**Affiliations:** 1 Center for Nutrition Research and Department of Nutrition, Food Science and Physiology, School of Pharmacy and Nutrition, University of Navarra, 31008 Pamplona, Spain; 2 Current Address: Center for Energy Metabolism and Reproduction, Shenzhen Institute of Advanced Technology, Chinese Academy of Science, Shenzhen, 518000 China; 3 Studies, Research and Sports Medicine Centre (CEIMD), Government of Navarre, 31005 Pamplona, Spain; 4 Centro de Investigación Biomédica en Red de Fisiopatología de la Obesidad y Nutrición (CIBEROBN), Instituto de Salud Carlos III (ISCIII), 28029 Madrid, Spain; 5 IdISNA, Navarra Institute for Health Research, 31008 Pamplona, Spain

**Keywords:** Overweight/obesity, post-menopause, MASLD, FOXO1, SIRT1, DHA, Exercise training, Aging

## Abstract

Sirtuins 1 (SIRT1) and Forkhead box protein O1 (FOXO1) expression have been associated with obesity and metabolic dysfunction-associated steatotic liver disease (MASLD). Exercise and/or docosahexaenoic acid (DHA) supplementation have shown beneficial effects on MASLD. The current study aims to assess the relationships between *Sirt1*, *Foxo1* mRNA levels and several MASLD biomarkers, as well as the effects of DHA-rich n-3 PUFA supplementation and/or exercise in the steatotic liver of aged obese female mice, and in peripheral blood mononuclear cells (PBMCs) of postmenopausal women with overweight/obesity. In the liver of 18-month-old mice, *Sirt1* levels positively correlated with the expression of genes related to fatty acid oxidation, and negatively correlated with lipogenic and proinflammatory genes. Exercise (long-term treadmill training), especially when combined with DHA, upregulated hepatic *Sirt1* mRNA levels. Liver *Foxo1* mRNA levels positively associated with hepatic triglycerides (TG) content and the expression of lipogenic and pro-inflammatory genes, while negatively correlated with the lipolytic gene *Hsl*. In PBMCs of postmenopausal women with overweight/obesity, *FOXO1* mRNA expression negatively correlated with the hepatic steatosis index (HSI) and the Zhejiang University index (ZJU). After 16-weeks of DHA-rich PUFA supplementation and/or progressive resistance training (RT), most groups exhibited reduced MASLD biomarkers and risk indexes accompanying with body fat mass reduction, but no significant changes were found between the intervention groups. However, in PBMCs n-3 supplementation upregulated *FOXO1* expression, and the RT groups exhibited higher *SIRT1* expression. In summary, SIRT1 and FOXO1 could be involved in the beneficial mechanisms of exercise and n-3 PUFA supplementation related to MASLD manifestation.

## Introduction

Non-alcoholic fatty liver disease (NAFLD) is commonly defined by the accumulation of neutral lipids to more than 5% of total liver weight, without alcohol over-consumption or other liver diseases [46]. Recently, the consensus has been reached to replace the nomenclature of NAFLD with metabolic dysfunction-associated steatotic liver disease (MASLD) [70]. MASLD encompasses various stages ranging from simple steatosis to metabolic dysfunction associated steatohepatitis (MASH). MASH can progress to cirrhosis, hepatocellular carcinoma and liver failure [64, 75, 89]. MASLD prevalence is increasing globally with aging, affecting 29.6% of the population in Asian countries [85], and around 17-45% of the general population in western countries [14]. Both obesity and aging contribute to the onset and progression of MASLD [31, 91]. In this sense, postmenopausal women with overweight/obesity may suffer from higher risk of developing MASLD than premenopausal individuals and age-matched men due to estrogen deficiency [8, 18, 22, 27].

Although the “golden standard” liver biopsy and conventional imaging techniques are valuable methods to diagnose MASLD in humans, they have many disadvantages, including invasiveness, sampling error, high cost, or risk of complications [77]. For this reason, several low cost and commonly available non-invasive indexes and blood biomarkers were developed and have been demonstrated to predict and diagnose MASLD with distinct precision [43, 92]. Among MASLD indexes, Fatty liver index (FLI), Hepatic steatosis index (HSI) and the Zhejiang University index (ZJU) have shown their diagnostic efficacy in multiple studies [10, 36, 45, 84].

Besides, some studies have brought out the use of whole blood RNA biomarkers or human peripheral blood mononuclear cell (PBMCs) gene expression to predict metabolic status or metabolic health [62, 68]. Several studies have suggested that Sirtuin 1 (SIRT1) and Forkhead box protein O1 (FOXO1) could play a role in lipid metabolism in obesity and MASLD in rodents [23, 61]. Furthermore, their plasma or PBMCs levels have been associated to obesity or MASLD in humans [41, 54]. The longevity-related SIRT1 may improve MASLD by regulating ROS, PGC-1α, FoxO1/3, and AMPK to restore mitochondrial function and reduce steatosis of the liver [35, 66, 71, 93]. SIRT1 has also been shown to downregulate hepatic *de novo* lipogenesis (*DNL*) [60, 65], while knocking out *Sirt1* in the liver of mice aggravated hepatic steatosis [66, 83]. Moreover, SIRT1 protects against systemic and liver inflammation through deacetylation and inhibition of Nuclear Factor Kappa B (NF-κB) transcriptional activity [66, 88].

FOXO1 seems to have a dual role in MASLD. On the one hand, hepatic FOXO1 overexpression promoted triglycerides (TG) accumulation, decreased fatty acid oxidation (FAO), and exacerbated MASLD progression in mice [56]. Besides, increased levels of FOXO1 were accompanied by endoplasmic reticulum (ER) stress activation in the liver of high fat diet (HFD)-induced MASH mice [26]. On the contrary, other studies have shown that transgenic mice overexpressing constitutively active FOXO1 (CA-FoxO1) in liver exhibit decreased fasting TG and cholesterol despite of hyperinsulinemia and suppressed hepatic *DNL* and *Srebp1*-c expression [24, 94, 95]. Similarly, isolated hepatocytes after the adenoviral expression of *Foxo1* increased TG turnover and FAO [24, 94, 95]. Moreover, liver specific *FoxO1/3/4* knock-out (LTKO) mice presented aggravated hepatic lipogenesis [24, 79] and increased expression levels of inflammatory and fibrotic genes when fed a HFD [61].

MASLD is associated with the depletion of n-3 PUFA and maintaining an optimally low ratio of n-6/n-3 PUFA was demonstrated to prevent hepatic steatosis and inflammation in MASLD [82]. Dietary supplementation with n-3 PUFA and physical exercise have been prompted as effective approaches to attenuate MASLD both in rodents [3, 81, 97] and humans [16, 28, 40, 44, 86]. A recent study of our group has found that long-term feeding with a docosahexaenoic acid (DHA)-enriched diet and/or exercise (treadmill) training could ameliorate liver steatosis in aged diet-induced obese (DIO) female mice, by upregulating FAO while decreasing lipogenic and inflammation-related genes [90]. Moreover, supplementation with DHA or with DHA-derived lipid mediators could prevent mitochondrial dysfunction, and reduce the inflammatory response and cellular damage [11, 59, 81].

Other study demonstrated that short-term DHA supplementation decreases hepatic steatosis and upregulates *Sirt1* mRNA and protein expression in the liver of middle-aged HFD-induced MASLD mice model [53]. Concerning the effects of n-3 PUFA on *Foxo1*, eicosapentaenoic acid (EPA) supplementation decreased hepatic steatosis and lowered hepatic FOXO1 protein expression in young Zucker rats [34]. Eight-week treatment with n-3 PUFA rearranged the highly expressed level of *Foxo1* in diabetic male Sprague Dawley rats [67].

The effect of exercise on the expression of *Sirt1* or *Foxo1* in the liver of rodents has also been investigated [9, 12, 15, 19, 50]. Both short (3-8 weeks) [12, 19, 50] and long-term (9 months) [9] treadmill exercise can induce SIRT1 activation. However, short-term high intensity training appeared to decrease *Foxo1* gene expression in the liver of lean male mice [15].

However, no studies have analysed the effect of long-term DHA rich n-3 PUFA supplementation and/or treadmill exercise on *Sirt1* and *Foxo1* expression in aged obese female mice and the possible mechanisms involved. Therefore, we hypothesize that *Sirt1* and *Foxo1* regulation could be related to the anti-inflammatory and anti-steatotic properties of long-term DHA rich n-3 PUFA supplementation and/or physical exercise (treadmill) that we have previously reported in aged obese female mice.

In humans, moderate physical activity time positively correlated with *SIRT1* and *FOXO1* mRNA expression in PBMCs in chronic obstructive pulmonary disease (COPD) patients [78].

Resistance training have been shown to reduce liver fat independent of weight loss in MASLD patients [33]. The effect of resistance exercise has been emphasized in decreasing the serum TG and aspartate aminotransferase (AST) levels in subjects with MASLD [73, 98]. To our knowledge, no study has evaluated the effect of DHA-rich n-3 PUFA supplementation and/or resistance training on *FOXO1* and *SIRT1* in PBMCs and their potential relationship with MASLD indexes and several serum metabolic biomarkers in humans. Therefore, we aimed to characterize if the expression levels of *FOXO1* and *SIRT1* in PBMCs in postmenopausal women with overweight/obesity are associated with MASLD risk biomarkers/indicators, and whether they can be modulated by DHA-rich n-3 PUFA supplementation and/or resistance training.

## Material and methods

### Animal study

Seven-week-old female C57BL/6 J mice purchased from Harlan Laboratories (Barcelona, Spain) were housed at the animal facilities of the University of Navarra under strict controlled conditions as mentioned previously [90]. All experiments were performed according to national animal care guidelines, and with the approval of the Ethics Committee for Animal Experimentation of the University of Navarra (Protocol 113–15), in accordance with the EU Directive 2010/63/EU. After acclimation, mice were divided into two experimental groups: (1) Control group fed a normal control diet containing as energy: 20% proteins, 67% carbohydrates, and 13% lipids, and (2) Diet- induced obese (DIO) group fed a high fat saturated diet (HFD) containing as energy: 20% proteins, 35% carbohydrates, and 45% lipids. Animals were fed ad libitum with these two diets for 4 months. Then, the DIO group was divided into 4 experimental groups: (1) DIO group that continued with the HFD up to 18 months; (2) DIO + DHA group fed with the HFD containing a DHA-rich n-3 PUFA concentrate from fish oil, replacing 15% wt/wt of dietary lipids up to 18 months; (3) DIO + Exercise (DIO + EX) group fed with the same HFD than the DIO group in combination with a treadmill exercise training up to 18 months; and (4) DIO + DHA + EX group fed with the HFD containing the DHA-rich n-3 PUFA concentrate, in combination with the treadmill training up to 18 months [90]. The control group was grown to 18 months as well. The DHA-rich n-3 PUFA concentrate (SOLUTEX0063TG, containing 683.4 mg DHA/g, 46.7 mg EPA/g, with a total content of n-3 PUFA of 838.9 mg/g as TG) was provided by Solutex, Spain. The different HFDs (prepared by Research Diets, Inc., New Brunswick, NJ, USA) were vacuum-sealed in 2.5 kg plastic bags, and kept frozen (− 20 ◦C) until used to avoid rancidity. The treadmill exercise program (LE8710M, Panlab, Barcelona, Spain) lasted from 6 until 18 months-old. Mice were allowed to adapt to the treadmill by running for 10 min on 2 consecutive days (first day at 3 m/min; second day at 4.8 m/min). Then, from months 6 to 10, mice were subjected to a low intensity training program (3 m/min for 5 min, increased to 4.8 m/min for 5 min, and then reached a maximum of 7.2 m/min for 20 min at 0% slope) during 3 alternate days per week. At 10 months of age (midlife), the number of sessions and the speed of the training were increased to 5 days per week during 5 weeks with the following protocol: running time and speed were started at 5 m/min for 5 min, increased to 8 m/min for 5 min, and then reached a maximum of 12 m/min for 20 min at 0% slope. During the next 7 months, the exercise protocol was maintained, but the number of sessions was reduced from 5 to 3 days a week. The mice of the non-exercise groups were left on the treadmill, without running, for the same period as the exercise groups [30, 90]. Mice were sacrificed after overnight fasting and liver were collected. Livers were weighed and kept at − 80 °C for further analysis.

### Human study design

This study encompasses an ancillary analysis of OBELEX study [29]. After thoroughly screened using an extensive medical history, resting electrocardiogram and blood pressure measurements, postmenopausal women aged between 55 and 70 years, with a BMI between 27.5 and 35 kg/m^2^, without changed weight in the last 3 months (± 3 kg) were enrolled by advertisement in local newspapers and by calls for volunteers from the database of the Metabolic Unit from the University of Navarra. Patients were excluded if they receive regular prescription medication, specially statins, antidiabetic drugs or hormone therapy; suffer from any chronic metabolic (severe dyslipidemia, type 1 or 2 diabetes), hepatic (cirrhosis), renal, cardiovascular, neuromuscular, arthritic, pulmonary, or other debilitating diseases; follow any special diets, such as Atkins, vegetarian, etc., prior 3 months the start of trial; have eating disorders or have been surgically treated of obesity or have alcohol or drug abuse. Participants were informed in detail about the possible risks and benefits of the project. The study was conducted with approval of the Research Ethics Committee of the University of Navarra (140/2015mod1 and 2015.140mod2) and was performed in compliance with the Helsinki Declaration guidelines. The study was registered at clinicaltrials.gov as NCT03300388. Written informed consent was collected from each of the subjects. 60 participants were included for current analysis. Participants were randomly categorized into four parallel groups for 16 weeks using the software platform MATLAB® (The Mathworks™, Natick, USA). Randomization criteria were age and BMI according to OMS classification. (1) Control group (P) received placebo capsules containing olive oil, (2) Omega-3 group (n-3) received DHA-rich n-3 PUFA capsules (55% DHA fish oil concentrate; Solutex, Madrid, Spain), providing 1.650 mg/day of DHA and 150 mg/day of EPA, (3) Resistance training group (P+RT) received placebo capsules and followed a progressive resistance training (PRT) program of 2 sessions/week, and (4) Omega-3 + Resistance training group (n-3+RT) received DHA-rich n-3 PUFA capsules and followed a PRT program of 2 sessions/week. All intervention groups received 6 capsules per day (2 per main meal) for the 16 weeks of intervention. At the end of the study, the adherence to treatment was evaluated by leftover pill count and depending on total attendance at the training sessions [7]. In addition, dietary advice for a healthy diet was provided during the whole trial to the four intervention groups, based on the dietary guidelines from the Spanish Society for Communitarian Nutrition [7]. Moreover, the consumption of fish was controlled depending on their n-3 PUFA’s composition according to Food Composition Tables [55] and online food composition databases (Easydiet® and Odimet®). At baseline and at the end of the trial, participants attended the Metabolic Unit at the University of Navarra in 8–12 h fasting conditions, where anthropometric measurements, body composition data were collected, and basal fasting blood samples were then extracted in order to obtain serum/plasma and PBMCs. Samples were kept at -80 ºC until analyses were performed. Every two weeks, volunteers met the dietitian to control weight and fat mass by bioimpedance and to ensure compliance of supplementation.

### Resistance training program

After the baseline visit was completed, subjects allocated in the RT groups were asked to assist to the Studies, Research, and Sports Medicine Center training facilities (CEIMD), with the collaboration of Dr. Javier Ibáñez-Santos, twice a week during 16 weeks of intervention, to perform dynamic resistance exercise [38, 39]. Eight exercises for upper and lower main muscular groups were included in the training program. Two routines were designed with six exercises each: leg press, chest press, knee extension and lat pulldown were maintained along the RT program, while shoulder press and hip extension (routine 1) and chest fly and leg curl (routine 2) were selected to complete each routine, changing every two weeks. Before testing and training, subjects attended three sessions for familiarization with the procedure of voluntary force production. A minimum of 2 days elapsed between two consecutive training sessions.

Strength tests were performed at the beginning, midst, and at the end of the trial to obtain strength gains/losses data and to adjust training loads to each volunteer’s strength. In this study, the 1-repetition maximum (1-RM) approach was used for testing [21]. Training progression was established using the pyramidal training approach, so as 50% of intensity was selected to start the training program, and a maximum intensity of 80% was reached at week 10 [5]. Three to four series were performed in each training session with 8-15 repetitions adapting to training loads. In each session, one of the researchers was present to direct and assist each subject towards ensuring adequate performance in each exercise (work rates, loads and ranges of motion) following American College of Sports Medicine (ACSM) guidelines for older adults. All subjects’ average compliance with the exercise sessions should be above 95% [29].

### Anthropometric assessments

Anthropometry measurements were performed in all participants. BMI was calculated in kg/m^2^; waist circumference was measured midway between the lower rib margin and the iliac crest. Changes in body composition were also analyzed at baseline and at the end of trial by Dual X-ray Absorptiometry (DXA) (Lunar iDXA, encore 14.5, Madison, WI, USA) [29].

### Serum biochemical assessment of liver function

Serum biochemical analyses were performed after 8-12 h fasting period. Serum glucose, total cholesterol, TG, alanine transaminase (ALT), AST, gamma-glutamyl transferase (GGT) levels were determined using a Pentra C200 autoanalyzer (Roche Diagnostic, Basel, Switzerland), following the manufacturer’s instructions.

### MASLD indexes calculation

FLI was calculated using BMI (kg/m^2^), serum TG (mg/dL) and GGT (U/L) concentrations and waist circumference (cm) according to Bedogni et al. [10] to obtain a score between 0 and 100: FLI < 30: no MASLD; 30 ≤ FLI < 60: Inconclusive; FLI ≥ 60: MASLD as following [10].

$$FLI = ({e}^{0.953*loge \left(TG\right)+ 0.139*BMI\;+\;0.718*loge\;\left(GGT\right)+\;0.053*waist\;circumference - 15.745})/(1+{e}^{0.953*loge\;(TG)\;+\;0.139*BMI\;+\;0.718*loge\;(GGT)\;+\;0.053*waist\;circumference - 15.745})*100$$

HSI was calculated using BMI (kg/m^2^), ALT (U/L), AST (U/L) concentrations, with the following formula [45]:

$$HSI=8\times ALT/AST+BMI\;(+\hspace{0.17em}2\;if\;diabetes,\hspace{0.17em}+\hspace{0.17em}2\;if\;female).$$

All participants were female and without diabetes. (HSI < 30: no MASLD; 30 ≤ HSI < 36: not classified; HSI ≥ 36: Highly likely to have MASLD).

ZJU was calculated basing on BMI, Fasting plasma glucose (FPG) (mmol/L), TG (mmol/L), ALT (U/L)/ AST (U/L) ratio, according to the following formula [84] :

$$ZJU=BMI+FPG+TG+3\times\;ALT/AST\;ratio\;(+\hspace{0.17em}2,\;if\;female).$$

ZJU < 32: Less likely to have MASLD; ZJU > 34: More likely to have MASLD in Chinese population.

### Analysis of mRNA Expression by Real-Time PCR

PBMCs were obtained in fasting conditions at baseline and at the end of the intervention as previously described [37]. Total RNA from PBMCs and from mice liver was extracted with TRIzol™reagent (Invitrogen, ThermoFisher Scientific). RNA quality and concentrations were measured by Nanodrop Spectrophotometer ND1000 (Nanodrop Technologies, Inc. Wilmington, NC, USA). RNA (5 μg) was then incubated with DNase I (Life Technologies, Carlsbad, CA, USA) for 30 min at 37 °C and reverse transcribed to cDNA using the High-Capacity cDNA Reverse Transcription Kit (Applied Biosystems; Thermo Fisher Scientific) according to the manufacturer’s instructions. Real-time PCR was performed using the Touch Real-Time PCR System (C1000 + CFX384, BIO-RAD, Hercules, CA, USA). The expression of genes was determined using Power SYBR Green PCR Master Mix (BIO-RAD) [90]. SYBR Green primers were obtained from published studies and tested with Primer-Blast software (National Center for Biotechnology Information, Bethesda, MD, USA; https://www.ncbi.nlm.nih.gov/tools/primer-blast): *Foxo1* mice (Forward primer: 5´- CTGCAGATCCCGTAAGACG -3´; Reverse primer: 5´-GGTCACCGGTGTCTAAGGAG -3´), *Sirt1* mice (Forward primer: 5´- TAGGGAACCTTTGCGTCATCT -3´; Reverse primer: 5´- CATTGTTGTTTGTTGCTTGGTC -3´), *FOXO1* human (Forward primer: 5´-CTGTGCGCCTGGACTCTTG-3´; Reverse primer: 5´-CAAGAGTCCAGGCGCACAG-3´), *SIRT1* human (Forward primer: 5´-GATCTTCCAGATCCTCAAGCG-3´; Reverse primer: 5´- AGGACATCGAGGAACTACCTG-3´) [57, 87]. 36B4 was used as the house-keeping gene [1]. Relative expression of the specific genes was determined using the 2^−ΔΔCt^ method [52].

### Statistical analysis

Statistical analyses were performed using SPSS Software, version 25 (IBM Corp.) and GraphPad Prism 9 software (Graph-Pad Software, La Jolla, CA, USA for mac OS). All data were expressed as means ± SD. Differences between groups were set up as statistically significant when *p* value was lower than 0.05. Once normality test and homogeneity tests of variances (Levene´s test) were conducted, comparisons between groups at baseline were analyzed by One-way ANOVA test or Kruskal-Wallis test. Comparisons between baseline and endpoint within each group were assessed by paired Student’s t test or Wilcoxon´s signed-rank test as appropriate. Two-way ANOVA test was performed to analyze whether the changes of dependent variable after intervention were caused by one of two treatments (independent factors): n-3 PUFA supplementation (n-3), RT or by an interaction between them (n-3×RT). If the statistical significance appeared at the interaction level (n-3xRT), multiple comparison post-hoc Tukey's HSD test were performed to differentiate the group effects. If not, the significant main effects were studied, which must be considered as (i) if *p* value < 0.05 appeared in the n-3 supplemented groups comparing with placebo groups, meaning that n-3 PUFA supplementation manifest the main effect of changing the dependent variable; (ii) if *p* value < 0.05 appeared in the RT groups comparing with non-RT groups, meaning that resistance training had the main effect of changing the dependent variable. Also, several analyses were carried out by adjusting potential covariates accordingly. Correlations were analyzed by either Pearson’s correlation for normally distributed variables or Spearman's correlation for not normally distributed variables.

## Results

### Expression levels of *Sirt1* and its correlation with proinflammatory and lipid metabolism related genes in liver of aged obese female mice

**Fig. 1:**
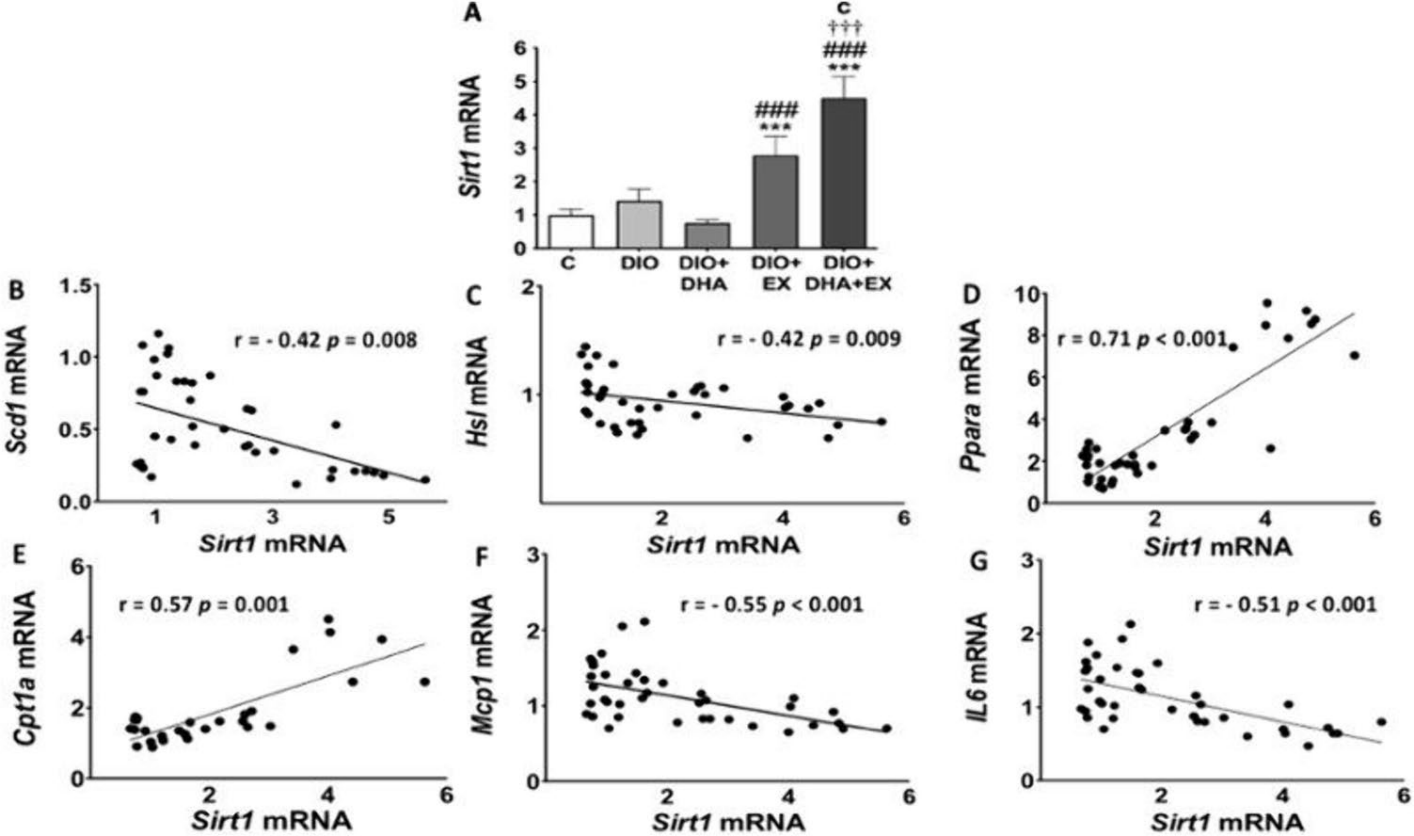
Effects of a long-term DHA-enriched HFD and/or exercise intervention on the mRNA expression level of *Sirt1* (**A**), and correlations between the expression levels of *Sirt1* and lipid metabolism-related genes (**B-E**) and pro-inflammatory genes (**F-G**) in the liver of aged obese female mice. Data are expressed as mean ± SD. *n* = 6–10. ^***^ *p* < 0.001 vs. C group; ^###^ *p* < 0.001 vs. DIO group; ^†††^ *p* < 0.001 for DIO + DHA vs. DIO + DHA + EX group; ^c^ *p* < 0.001 for DIO + EX vs. DIO + DHA + EX group. Correlation analyses were carried out using Pearson or Spearman correlation coefficients after testing for normality

As previously mentioned, SIRT1 has been identified as a protector of liver inflammation and a regulator of hepatic lipid metabolism [25]. Here, *Sirt1* mRNA expression did not manifest significant differences between control group and DIO group. But interestingly, treadmill exercise alone or especially when combined with the DHA-enriched diet significantly potentiated *Sirt1* expression in liver of obese aged mice. No significant effects were found for DHA supplementation alone (Figure 1A).

In order to determine whether *Sirt1* mRNA expression was associated with liver TG content or the expression of genes determining hepatic fat accumulation or inflammation, correlation analyses were carried out. Our data showed that *Sirt1* was not correlated with liver TG content (r = -0.07, *p* = 0.68). However, *Sirt1* expression negatively correlated with *Scd1* expression (Figure 1B), which participates in the *DNL* and converts saturated fatty acids to monounsaturated fatty acids [42, 74]. Although a negative correlation was found between *Sirt1* and the expression level of the lipolytic gene *Hsl* (Figure 1C), *Sirt1* mRNA levels were positively correlated with some genes involved in FAO such as *Ppara* and *Cpt1a* (Figures 1D, E). As a protector of liver inflammation [32], *Sirt1* expression showed a negative correlation with pro-inflammatory genes including *Il6* and *Mcp1* (Figures 1F, G). Moreover, a marginally negative association was found between *Sirt1* and *Tnfa* expression (r = -0.29, *p* = 0.08) in fatty liver of aged obese female mice.

### Expression levels of *Foxo1* and its correlation with liver TG, proinflammatory and lipid metabolism related genes in liver of aged obese female mice

**Fig. 2:**
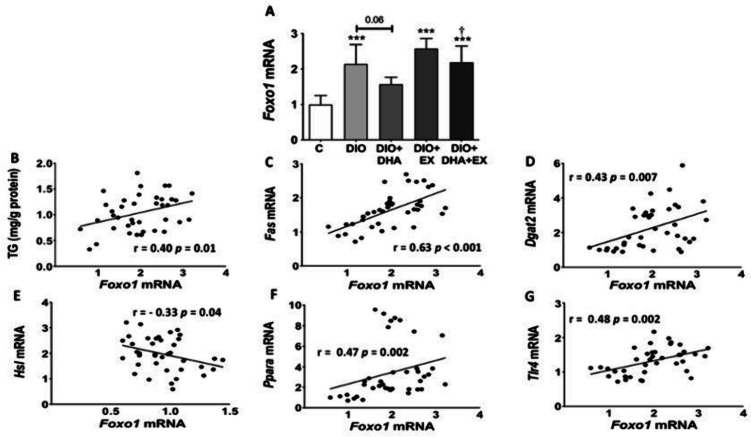
Effects of a long-term DHA-enriched HFD and/or treadmill exercise intervention on the mRNA expression level of *Foxo1* (**A**), and correlations between the expression levels of *Foxo1* and liver triglyceride (TG) content (**B**) and lipid metabolism-related genes (**C-F**) and pro-inflammatory genes (**G**) in the liver of aged obese female mice. Data are expressed as mean ± SD. *n* = 6–10. ^***^ *p* < 0.001 vs. C group; ^†^ *p* < 0.05 for DIO + DHA vs. DIO + DHA + EX group. Correlation analyses were carried out using Pearson or Spearman correlation coefficients after testing for normality

FOXO1 is a transcriptional target of SIRT1 [13] and has been suggested to be involved in hepatic lipid metabolism regulation [48] and inflammation [51]. Liver *Foxo1* mRNA expression level was upregulated by HFD (Figure 2A). Exercise did not induce any relevant change on *Foxo1* mRNA levels. However, the chronic feeding with the DHA-enriched diet showed a marginally significant tendency to downregulate the increased level of *Foxo1* induced by the HFD. However, this trend was not observed when DHA was combined with exercise (Figure 2A).

Furthermore, a positive association between *Foxo1* mRNA expression and hepatic TG content was found in the liver of 18 months old female mice (Figure 2B). In parallel with this, *Foxo1* expression positively correlated with the expression of the lipogenic genes *Fas* and *Dgat2* (Figures 2C, D), and negatively correlated with the expression of the lipolytic gene *Hsl* (Figure 2E). Unexpectedly, a positive association was also found between the expression of *Foxo1* and *Ppara* (Figure 2F). On the other hand, *Foxo1* expression was positively associated with the pro-inflammatory gene *Tlr4* expression (Figure 2G), although no significant correlations were found between the expression of *Foxo1* and other pro-inflammatory genes such as *Il6*, *Mcp1* and *Tnfa* (data not shown).

### *FOXO1* and *SIRT1* mRNA expression in PBMCs and MASLD indexes in postmenopausal women with overweight/obesity

Next, we aim to evaluate *FOXO1* and *SIRT1* mRNA levels in PBMCs in a cohort of postmenopausal overweight/obese women and the potential association with MASLD indexes. Basal characteristics of the study subjects are shown in Table 1. The mean serum values of AST, ALT and GGT were in the non-pathological stage. Several MASLD indexes were calculated in order to estimate the presence/risk of MASLD in these postmenopausal women with overweight/obesity (Table 1). According to the FLI criteria only 19 of them would meet the criteria for diagnosing MASLD. However, based on the HSI and ZJU indexes, 56 and 60 of them would meet the MASLD criteria, respectively.

**Table 1:** Baseline characteristics of participants, MASLD indexes and correlation with PBMCs *FOXO1* and *SIRT1* mRNA expression

Variable	*N*	*SIRT1 mRNA*		*FOXO1* mRNA	
	60	r	*p* value	r	*p* value
Age	58.7 ± 3.2	0.07	0.61^b^	-0.09	0.49^b^
BMI (kg/m^2^)	30.6 ± 2.0	-0.04	0.78^b^	-0.25	0.056^b^
Fat Mass (kg)	36.4 ± 4.6	-0.004	0.97^a^	0.13	0.33^b^
Waist Circumference (cm)	93.8 ± 5.9	-0.05	0.70^a^	-0.12	0.38^b^
Glucose (mmol/L)	5.8 ± 0.9	-0.21	0.11^b^	-0.08	0.50^b^
TG (mg/dL)	106.0 ± 44.7	-0.08	0.55^b^	-0.08	0.60^b^
Cholesterol (mg/dL)	250.5 ± 36.5	0.16	0.24^b^	0.28	**0.03**^b^
AST (U/L)	20.5 ± 5.9	-0.24	0.06^a^	-0.2	0.1^b^
ALT (U/L)	19.1 ± 9.6	-0.25	0.06^b^	-0.25	0.05^b^
GGT (U/L)	21.8 ± 21.7	-0.15	0.26^b^	-0.13	0.31^b^
FLI	48.2 ± 19.5	-0.13	0.31^b^	-0.24	0.06^b^
HSI	39.9 ± 3.1	-0.19	0.15^a^	-0.34	**0.008**^b^
ZJU	40.4 ± 2.6	-0.13	0.33^a^	-0.27	**0.04**^b^

Correlation analysis was studied by ^a^Pearson Correlation Coefficients or ^b^Spearman Correlation Coefficients after testing for normality. Data are expressed as mean ± SD

In order to evaluate whether there is a potential association between these MASLD markers/indexes and *FOXO1* or *SIRT1* mRNA expression in PBMCs, correlation analyses were carried out. Our data show that no correlation was found between *SIRT1* and the MASLD markers, although a tendency to negatively correlate with serum ALT (r = -0.25, *p*= 0.06) and AST (r = -0.24, *p*= 0.06) levels was found (Table 1).

However, *FOXO1* mRNA expression showed a significant negative correlation with HSI and ZJU index (Table 1). In addition, *FOXO1* showed a tendency to negatively correlate with BMI, serum ALT and FLI index (Table 1). Surprisingly, a positive correlation was found for *FOXO1* mRNA expression and serum cholesterol levels.

### Effects of DHA rich n-3 PUFA supplementation and/or Resistance training on MASLD indexes

We next aim to characterize the effects of a 16-week intervention with a DHA-rich n-3 PUFA supplementation alone or in combination with a RT program on MASLD indexes in postmenopausal women with overweight/obesity.

Overall, all study groups showed a significant reduction in body fat mass after the intervention as compared with baseline values. However, the analysis of differences of changes between groups by 2-way ANOVA revealed no significant differences by either DHA-rich n-3 PUFA supplementation or RT (Table 2), in agreement with our previous published data [29]. Waist circumference was significantly reduced in all groups except the RT group, but no significant differences between groups were found. ALT and GTT also tended to be reduced within all experimental groups, but significant differences were only found for ALT in the P group and for GTT in the n-3 group at the end of the intervention. However, the analysis of the differences in changes between groups revealed no significant changes (Table 2).

**Table 2:** Effects of 16-week intervention with a DHA-rich n-3 PUFA supplementation and/or RT program on anthropometric, biochemical parameters and MASLD indexes in postmenopausal women with overweight/obesity

	P	n-3	P + RT	n-3 + RT	Two-way ANOVA		
N	15	14	16	15	n-3	RT	n-3 × RT
BMI (kg/m^2^)							
Baseline	30.6 ± 2.3	30.1 ± 1.8	30.7 ± 2.3	31.0 ± 1.9			
Change	-1.0 ± 1.1	-1.1 ± 1.0	-0.9 ± 1.0	-1.1 ± 1.4	ns	ns	ns
Body fat mass (kg)							
Baseline	35.2 ± 5.1	36.6 ± 4.1	37.9 ± 4.4	35.7 ± 4.7			
Change	-3.5 ± 2.6^***^	-2.6 ± 2.5^**^	-2.7 ± 2.8^**^	-2.2 ± 1.6^**^	ns	ns	ns
Waist C (cm)							
Baseline	93.7 ± 3.7	94.3 ± 7.4	93.3 ± 5.0	94.1 ± 7.4			
Change	-1.8 ± 1.5^**a^	-2.0 ± 1.8^**^	-0.03 ± 3.9	-1.9 ± 3.4^*^	ns	ns	ns
Glucose (mmol/L)							
Baseline	5.4 ± 0.7	5.7 ± 0.8	6.1 ± 1.1	6.0 ± 0.6			
Change	-0.1 ± 0.5	-0.1 ± 0.7	-0.2 ± 1.2	-0.3 ± 0.5	ns	ns	ns
Cholesterol (mg/dL)							
Baseline	245.6 ± 25.8	240.8 ± 48.0	258.0 ± 30.0	256.4 ± 40.3			
Change	-15.4 ± 23.5^*a^	-11.0 ± 45.9	-21.6 ± 26.9^*a^	-17.7 ± 42.7	ns	ns	ns
ALT (U/L)							
Baseline	20.2 ± 11.0	19.4 ± 10.1	16.7 ± 6.9	20.2 ± 10.6			
Change	-2.7 ± 5.2^*a^	-0.5 ± 5.8	-2.2 ± 4.7	-0.7 ± 7.7	ns	ns	ns
AST (U/L)							
Baseline	22.3 ± 4.5	20.1 ± 6.7	18.7 ± 3.5	20.9 ± 7.9			
Change	-1.1 ± 3.3	1.0 ± 6.5	-0.9 ± 3.4	0.4 ± 6.9	ns	ns	ns
GGT (U/L)							
Baseline	16.1 ± 9.3	32.9 ± 37.9	15.8 ± 6.4	23.8 ± 17.9			
Change	-1.5 ± 2.9	-5.8 ± 9.2^*a^	-0.8 ± 4.0	-1.6 ± 7.1	ns	ns	ns
FLI							
Baseline	41.3 ± 17.0	53.3 ± 21.1	47.2 ± 18.7	51.6 ± 20.8			
Change	-6.5 ± 8.2^*a^	-13.6 ± 10.2^***^	-7.0 ± 12.0^*^	-10.4 ± 12.4^**^	0.07	ns	ns
HSI							
Baseline	39.7 ± 3.5	39.8 ± 3.1	39.7 ± 3.1	40.6 ± 2.7			
Change	-1.5 ± 1.3^***^	-1.6 ± 1.9^***^	-1.1 ± 2.6	-1.3 ± 2.4	ns	ns	ns
ZJU							
Baseline	39.7 ± 3.1	40.0 ± 2.5	40.8 ± 2.5	41.0 ± 2.5			
Change	-1.3 ± 1.3^**^	-1.7 ± 1.6^**^	-1.3 ± 1.9^*^	-1.6 ± 1.9^*^	ns	ns	ns

Baseline-endpoint differences were studied by paired Student’s t-test or ^a^Wilcoxon’s signed-rank test after testing for normality. Differences in changes between groups were compared by two-way ANOVA. Data are expressed as mean ± SD. *n* = 14–16. ^*^p < 0.05. ^**^ *p* < 0.01, ^***^ *p* < 0.001. *ns* nonsignificant

Regarding MASLD indexes, FLI and ZJU index showed a significant reduction in all experimental groups at the end of the intervention. HSI was also decreased in all experimental groups, but the reduction was not significant in RT and n-3 + RT groups. However, the two-way ANOVA revealed no significant changes between groups for any of the MASLD indexes analysed. The only remarkable observation was a trend (*p* = 0.07) for FLI to be reduced in the n-3 supplemented groups (Table 2). Similar effects were observed when the changes were adjusted by body fat mass losses (data not shown).

### Resistance training upregulates *SIRT1* mRNA expression in PBMCs from postmenopausal women with overweight/obesity

Finally, we aimed to study the effects of a 16-week intervention with a DHA-rich n-3 PUFA supplementation alone or in combination with a RT program on the changes of *SIRT1* and *FOXO1* mRNA expression in PBMC from postmenopausal women with overweight/obesity.

**Fig. 3:**
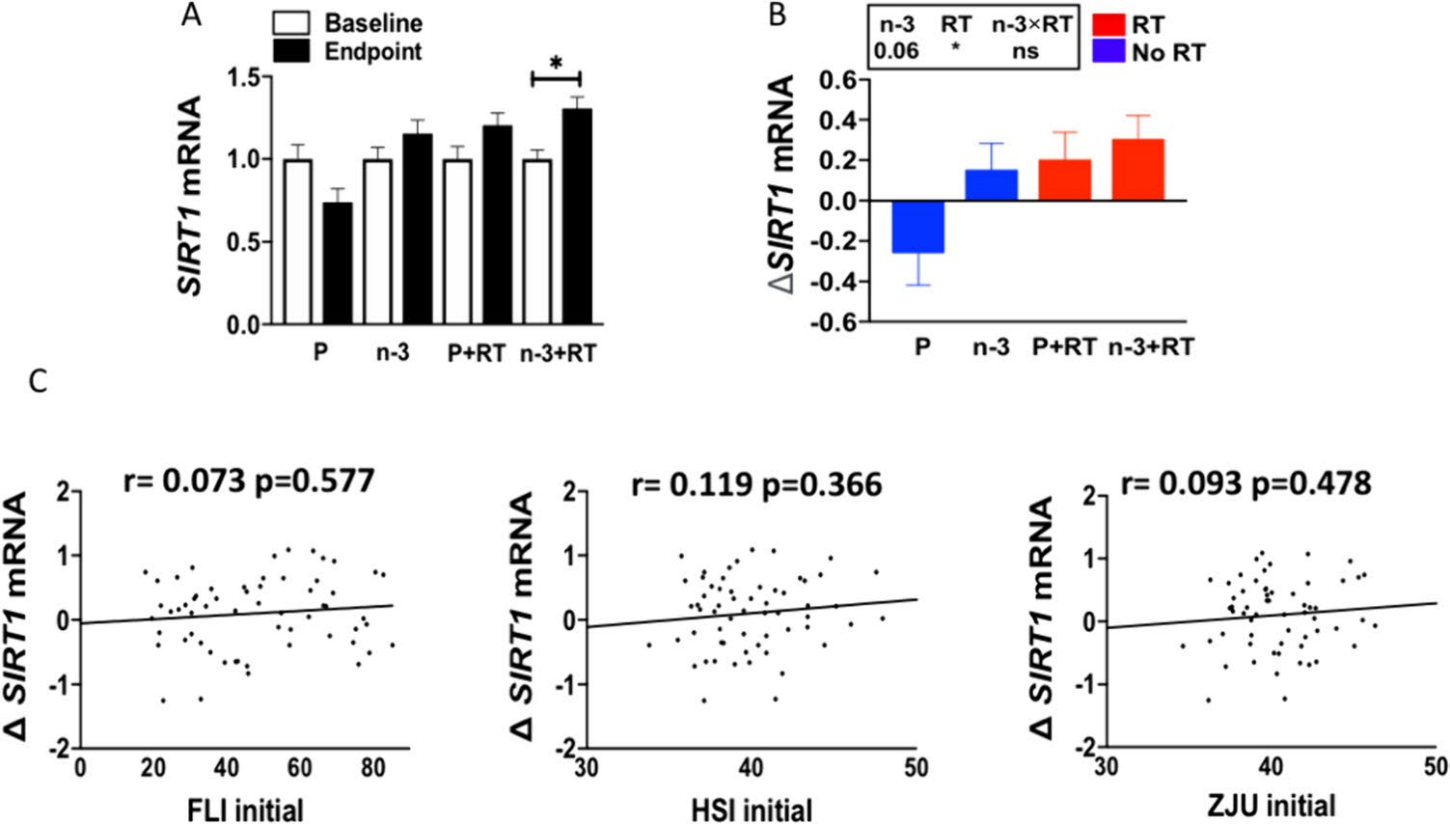
Effects of 16-week intervention with a DHA-rich n-3 PUFA supplementation and/or RT program on *SIRT1* mRNA expression in PBMCs (**A-B**) and correlation between changes of *SIRT* expression and baseline MASLD indexes (**C**) in postmenopausal women with overweight/obesity. Baseline-endpoint differences were studied by paired Student’s t-test or Wilcoxon’s signed-rank test after testing for normality. Differences in changes between groups were compared by two-way ANOVA. Data are expressed as mean ± SD. *n* = 14–16 per group. ^*^p < 0.05. *ns* nonsignificant

Figure 3A shows that *SIRT1* mRNA expression was statistically upregulated in the n-3 + RT group at the endpoint (*p* < 0.05). A non-significant trend was also observed in the P+RT group. Accordingly, the analyses of the differences in changes between groups (by two-way ANOVA) revealed that the RT intervention (RT and n-3 + RT) had a significant (*p* < 0.05) main effect in elevating *SIRT1* mRNA expression as compared to the non-trained groups (Figure 3B). Meanwhile, DHA rich n-3 PUFA supplementation (n-3, n-3 + RT groups) showed a tendency to upregulate the *SIRT1* mRNA expression (*p* = 0.06). No interaction effect of DHA rich n-3 PUFA supplementation and resistance training were detected on *SIRT1* mRNA expression (Figure 3B).

No significant correlations were observed between baseline FLI, HSI, ZJU index and changes in *SIRT1* mRNA expression (Figure 3C). In addition, no apparent association between MASLD indexes changes and *SIRT1* mRNA expression changes were found (data not shown).

### DHA rich n-3 PUFA supplementation increases *FOXO1* mRNA expression in PBMCs of postmenopausal women with overweight/obesity

**Fig. 4:**
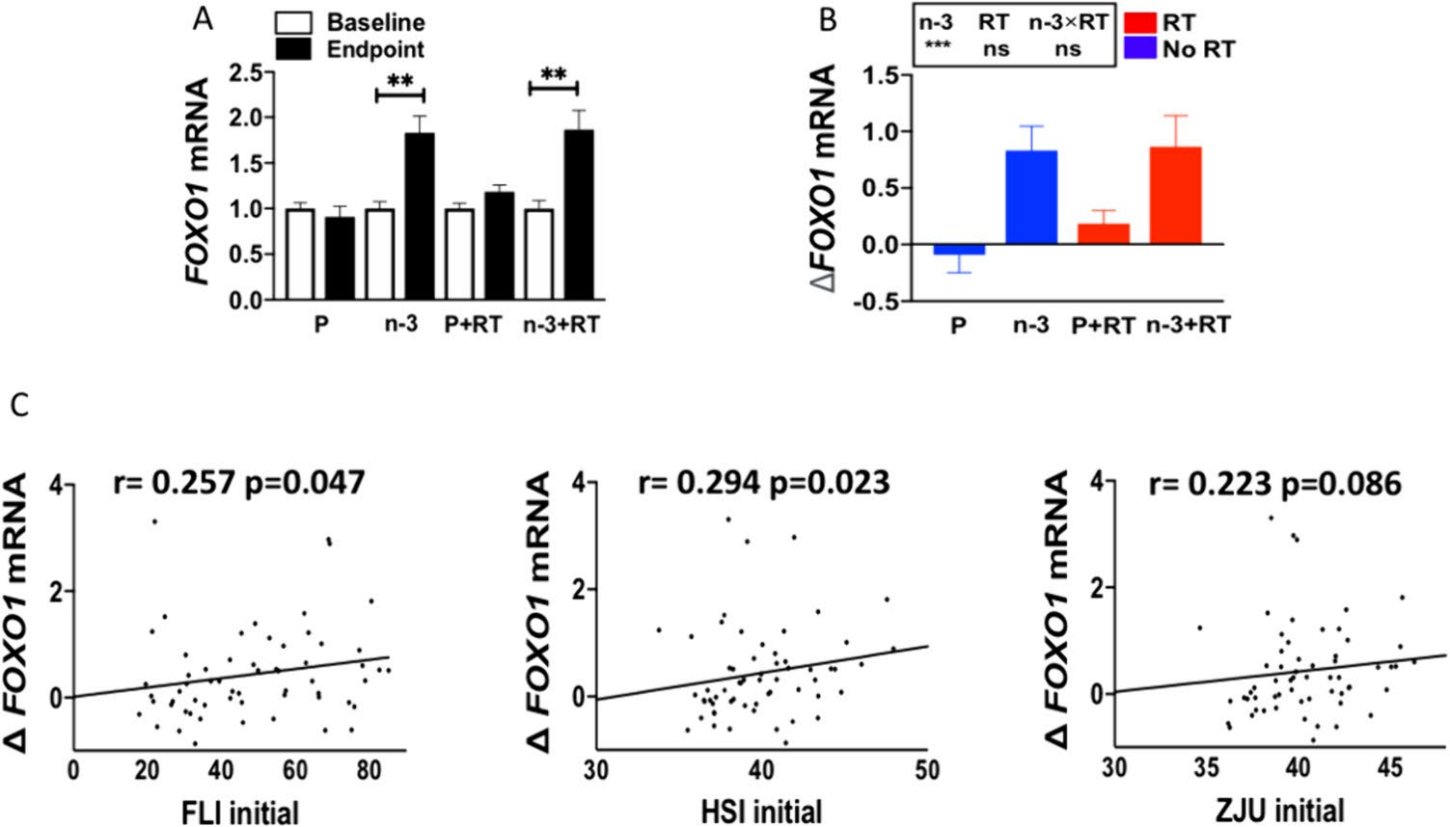
Effects of 16-week intervention with a DHA-rich n-3 PUFA supplementation and/or RT program on *FOXO1* mRNA expression in PBMCs (**A-B**) and correlation between changes of *FOXO1* expression and baseline MASLD indexes (**C**) in postmenopausal women with overweight/obesity. Baseline-endpoint differences were studied by paired Student’s t-test or Wilcoxon’s signed-rank test after testing for normality. Differences in changes between groups were compared by two-way ANOVA. Data are expressed as mean ± SD. *n* = 14–16 per group. ^**^ *p* < 0.01, ^***^ *p* < 0.001. *ns* nonsignificant

As shown in Figure 4A, *FOXO1* mRNA expression was significantly increased within the DHA rich n-3 PUFA supplemented groups at the end of 16-weeks of intervention (*p* < 0.01) as compared with baseline. No significant changes were found in the P and P+RT groups. In agreement, the analysis of the changes (endpoint-baseline) between groups showed that the DHA rich n-3 PUFA supplementation (n-3 and n-3 + RT groups) had a significant effect in upregulating *FOXO1* mRNA expression (*p* < 0.001) as compared to the placebo supplemented groups (P and P+RT). No interaction effect of DHA rich n-3 PUFA supplementation and resistance training was detected (Figure 4B).

Moreover, the correlation analyses showed that the changes in *FOXO1* mRNA expression positively correlates with baseline FLI and HSI values. Only a tendency was found for the ZJU index (Figure 4C). These data suggest that initial FLI and HSI index were also sensible to predict the changes of *FOXO1* mRNA expression. However, no correlation was found between the changes observed in the *FOXO1* expression and the changes induced by the intervention on FLI, HSI and ZJU index (data not shown).

## Discussion

This study evaluated if the beneficial effects of long-term DHA supplementation alone or combined with physical exercise could be related with changes in the expression of *Sirt1* and *Foxo1*. SIRT1 and FOXO1 have shown to play a role in lipid metabolism in obesity and MASLD conditions [23, 54, 61]. The longevity-related factor SIRT1 [60] has also been found to downregulate hepatic *DNL* [65], and liver specific *Sirt1* knockout mice manifested hepatic steatosis [83].

Our current data show that hepatic *Sirt1* mRNA expression did not manifest statistically significant differences between control group and the DIO group in 18 month-old female mice. However, Luo et al.  [53] have reported a reduced level of *Sirt1* expression in 9-month male mice fed with HFD for 5 months. Consistent with this, Chyau et al. [20] showed a decreased level of SIRT1 protein expression in 6-week-old male mice fed with HFD for 2 months. However, Liou et al. [49] found that feeding 4 weeks old male mice with HFD for 4 months increased the protein expression level of SIRT1. Interestingly, our study revealed that treadmill exercise alone or especially when combined with DHA-enriched diet significantly potentiated hepatic *Sirt1* expression in aged DIO mice. This upregulation of *Sirt1* by long-term treadmill exercise was consistent with previous investigations conducted in young or middle-aged rodents with short/long-term treadmill training [9, 12, 19, 50]. Nevertheless, 8 weeks treadmill running did not induce significant increase in hepatic SIRT1 expression in HFD-induced MASLD male rat [58]. In our model of old obese female mice, DHA rich n-3 PUFA supplementation alone did not have any significant improvement on *Sirt1* expression. However, Luo et al. [53] found that 8 weeks of DHA supplementation decreased liver lipid accumulation, increased FAO and upregulated hepatic *Sirt1* in middle-aged DIO mice. Moreover, DHA also prevented the reduction of *Sirt1* levels induced by palmitic acid (PA) in Hep-G2 cells. Interestingly, *Sirt1* knock-down or inhibition prevented the beneficial effect induced by DHA *in vivo* and *in vitro* [53]. The lack of remarkable effects of DHA supplementation alone on *Sirt1* expression observed in our study may possibly be due to the more advanced age of these animals as compared to the previously mentioned studies. However, it is important to highlight that DHA rich n-3 PUFA supplementation strongly potentiated the stimulatory effect of exercise on the upregulation of *Sirt1* in liver of aged DIO mice. This finding is an interesting outcome and to our knowledge no previous studies have investigated the effect of combining exercise with n-3 PUFA on the expression of *Sirt1* in mice liver.

Noteworthily, a negative correlation between the expression of *Sirt1* and the mRNA levels of the lipogenic gene (*Scd1*) was observed. In parallel, a positive correlation was found between the expression of *Sirt1* and FAO-related genes (*Ppara* and *Cpt1a*). Furthermore, *Sirt1* had a negative correlation with pro-inflammatory genes (*Il6* and *Mcp1*). In line with the negative correlation between *Sirt1* and *Scd1*, Sharma et al. [76] showed that treatment with Barbamine, a compound that activated the SIRT1/LKB1/AMPK pathway prevented the elevation in the expression of FAS and SCD1 in the liver of rats with MASLD induced by HFD. Moreover, other studies administrating chemical activators of SIRT1, resveratrol and SRT1720, or the NAD+ precursor nicotinamide riboside, reduced SREBP1c, FAS, SCD1 gene and protein expression in the liver of mice/rats with HFD-induced hepatic steatosis [2, 6, 65, 76]. It has been also described that SIRT1 activation can increase FAO by deacetylating PPARα/PGC-1α [66, 93]. SIRT1 overexpression using adenovirus can reduce PGC-1α acetylation level and induced PPARα transcriptional signalling, as well as the expression of PPARα/PGC-1α targeting genes, thereby increasing FAO, and alleviating MASLD in mice with HFD-induced hepatic steatosis [66, 72]. Moreover, Purushotham et al. [66] demonstrated that SIRT1 deficiency caused decreased level of *Cpt1*, which further supports our observation. Additionally, Pfluger et al. [63] suggested that transgenic mice model overexpressing *Sirt1* exhibited less levels of pro-inflammatory biomarkers (IL-6 and TNF-α), mediated by downmodulation of NFκB activity. This seems to coincide with our outcomes, showing that *Sirt1* negatively correlates with the hepatic expression level of *Il6* and *Mcp1.* Therefore, to some extent, it is likely that *Sirt1* activation could be mediating the beneficial effects of exercise alone and especially in combination with n-3 PUFA supplementation on the prevention of MASLD/MASH associated to obesity and aging.

Our data evidenced that *Foxo1* mRNA levels were increased in the aged DIO mice and positively correlated with hepatic TG content. Moreover, a positive association between *Foxo1* and lipogenic gene expression (*Fas* and *Dgat2*), and the expression of the pro-inflammatory gene *Tlr4* was found in the liver of aged obese mice. Furthermore, *Foxo1* negatively correlated with the expression of the lipolytic gene (*Hsl*). These data suggest that *Foxo1* may favour the progression of MASLD by aggravating hepatic steatosis and inflammation in the liver of aged obese female mice. This is in line with the study of Ding et al. [26] showing increased levels of FOXO1 in the liver of mice with HFD-induced MASH. On the other hand, DHA supplementation demonstrated a tendency to decrease *Foxo1* expression, but no significant changes were observed either with DHA or with exercise alone or in combination. However, Hong et al. [34] demonstrated that 8-weeks of EPA supplementation significantly decreased FOXO1 protein expression and hepatic steatosis in young obese male Zucker rats. In consistence with this, Chen et al. [17] indicated that 30 days of DHA supplementation downregulated gene and protein expression of FOXO1 in the liver and adipose tissue of weaned pigs. Concerning the effects of exercise on FOXO1, Castaño et al. [15] showed that short-term high intensity training decreased hepatic *Foxo*1 expression in male mice. These data suggest that the downregulation of hepatic *Foxo1* could participate in the beneficial effects of n-3 PUFAs and exercise on MASLD. The lack of significant effects of DHA supplementation and/or exercise on hepatic *Foxo1* expression observed in our study could be related with the more advanced age of our mice model as compared with the previous studies, and to the fact that our study was performed in female mice, while most of the previous studies were carried out in male mice.

In postmenopausal women with overweight/obesity, our data displayed no significant association between the expression level of *SIRT1* in PBMCs and MASLD biomarkers/indexes. Concerning *FOXO1*, and in contrast to what was observed in the liver of aged obese female mice, a negative correlation between *FOXO1* mRNA expression in PBMCs and MASLD risk markers (HSI and ZJU) was observed. Although surprisingly, a positive correlation between the expression of *FOXO1* and serum cholesterol levels was also found. Clinical trials analysing the expression levels of SIRT1/FOXO1 in MASLD/MASH patients and their associations with liver fat accumulation are scarce. In this way, Valenti et al. [80] showed higher levels of hepatic FOXO1 mRNA and protein expression levels in MASH patients compared to patients with hepatic steatosis and normal patients. Moreover, they indicated a positive correlation between *FOXO1* mRNA levels and MASH activity score, which contrast with our observation in PBMCs. Another study found an upregulated level of *FOXO1* expression in the PBMCs of obese patients as compared to normal-weight subjects [41]. Concerning SIRT1, a trial conducted in obese patients with MASLD showed a lower plasma SIRT1 levels in patients with either moderate or severe liver steatosis compared to the mild steatosis group, and that plasma SIRT1 negatively correlated with liver steatosis [54].

The lack of correlation between the expression level of *SIRT1* in PBMCs and MASLD biomarkers/indexes, and the apparently inconsistent result observed in the expression level of *FOXO1* in PBMCs in our study and the study of Valenti et al. [80] in liver biopsies in 27 patients with MASH, can be explained by the fact that our participants were apparently healthy postmenopausal women with overweight/obesity and were not clinically diagnosed with MASLD/MASH, and based on the index used some of them might have developed early stages of MASLD (liver steatosis).

Moreover, we investigated the effect of a 16-week DHA rich n-3 supplementation and/or a progressive resistance training on MASLD indexes. To our knowledge, no study has evaluated the effect of DHA rich n-3 PUFA supplementation and/or resistance training on *FOXO1* and *SIRT1* expression in PBMCs in postmenopausal women with overweight/obesity and their potential relationship with MASLD indexes. We evidenced that 16 weeks of DHA rich n-3 supplementation and resistance training alone, or in combination ameliorated serum MASLD biomarkers (AST, ALT, GGT) and MASLD risk indexes (FLI, HSI and ZJU) in parallel with a reduction of body fat mass, but to a similar extent than the placebo group. These observations can be attributed to the dietary advice for a healthy diet received by all intervention groups [29], and the anti-steatotic effect of all interventions. Indeed, the placebo used in our study is olive oil (6 capsules of 0.5 g olive oil each), and it has been shown that olive oil (20 g/d for 12 weeks) consumption can lower fatty liver grade, serum AST, serum TG and body fat mass [69].

An interesting finding is that resistance training induced an upregulation of *SIRT1* expression in PBMCs, while no correlations were found between *SIRT1* changes and changes or initial levels of MASLD indexes. This suggests that the induction of *SIRT1* could be a mechanism participating also in other metabolic health benefits induced by resistance training exercise. In this context, Taka et al. [78] analyzed *SIRT1, FOXO1* mRNA expression in PBMCs of patients with chronic obstructive pulmonary disease (COPD) and demonstrated that *SIRT1* and *FOXO1* mRNA expression levels were positively correlated with moderate-physical activity time.

We also found that DHA-rich supplementation induced a significant upregulation of *FOXO1* expression as compared to the placebo supplemented groups. Moreover, changes in *FOXO1* expression were positively correlated with baseline FLI, HSI indexes, indicating that initial FLI and HSI index are sensitive to predict the changes of *FOXO1* expression. To our knowledge, no previous studies have demonstrated the upregulating effect of DHA rich n-3 PUFA supplementation on PBMCs *FOXO1* expression or the correlation with MASLD indexes in humans. These results contrast with some preclinical studies in different animal models, showing reductions in FOXO1 in liver after n-3 PUFA supplementation [17, 34]. Concerning some limitations of the study, it would be also of interest to carry out age-matched studies in overweight/obese men to characterize if the outcomes were similar to those observed in postmenopausal women. Additionally, it would have been of interest to assess the effects of the intervention on the expression levels of pro-inflammatory genes in PBMCs, since previous studies have demonstrated the beneficial effects of n-3 PUFA supplementation in alleviating the level of inflammatory markers such as IL-6, TNF-α in human plasma/serum [4, 47].

In summary, our data in mice reveal negative correlations between *Sirt1* and genes involved in hepatic lipid accumulation and inflammation, as well as a *Sirt1* upregulation induced by treadmill exercise, especially when combined with DHA-rich diet, suggesting that *Sirt1* could participate in the beneficial mechanisms of exercise to alleviate MASLD. Interestingly, resistance training also upregulates *SIRT1* expression in PBMCs of postmenopausal women with overweight/obesity, but no correlation has been found with changes in MASLD indexes. Additionally, obese aged mice exhibit increased levels of hepatic *Foxo1*; however, in postmenopausal women with overweight/obesity *FOXO1* in PBMCs negatively correlates with MASLD indexes and DHA-rich n-3 PUFA supplementation upregulates *FOXO1*. Therefore, due to the dual role described for *FOXO1* on MASLD development, future studies in large cohorts of well-diagnosed patients with MASLD/MASH are needed to establish if *FOXO1* expression in PBMCs could be a biomarker of these pathologies and of the response to dietary/pharmacological lifestyle interventions.

## Data Availability

The data that support the findings of this study could be available from the corresponding author upon reasonable request.
